# Supplementary material for: Functional synaptic connectivity shapes spine stability in the hippocampus
Source: Nat Commun. 2026 Apr 4;17:3218. doi: 10.1038/s41467-026-71332-z (PMC13057201; doi:10.1038/s41467-026-71332-z)
Supplement: Supplementary file 1 — Supplementary Information [file 41467_2026_71332_MOESM1_ESM.pdf]

Functional Synaptic Connectivity Shapes Spine Stability in the Hippocampus

Cynthia Rais[1] and J. Simon Wiegert[1,2,*]

Affiliations:

[1]Research Group Synaptic Wiring and Information Processing, Center for Molecular Neurobiology Hamburg, University Medical Center Hamburg-Eppendorf, Hamburg, Germany

[2]Department of Neurophysiology, Medical Faculty Mannheim, MCTN, Heidelberg University, Mannheim, Germany

* Correspondence to: simon.wiegert@medma.uni-heidelberg.de

Supplementary Information:

Figures S1- S9

Supplemental References

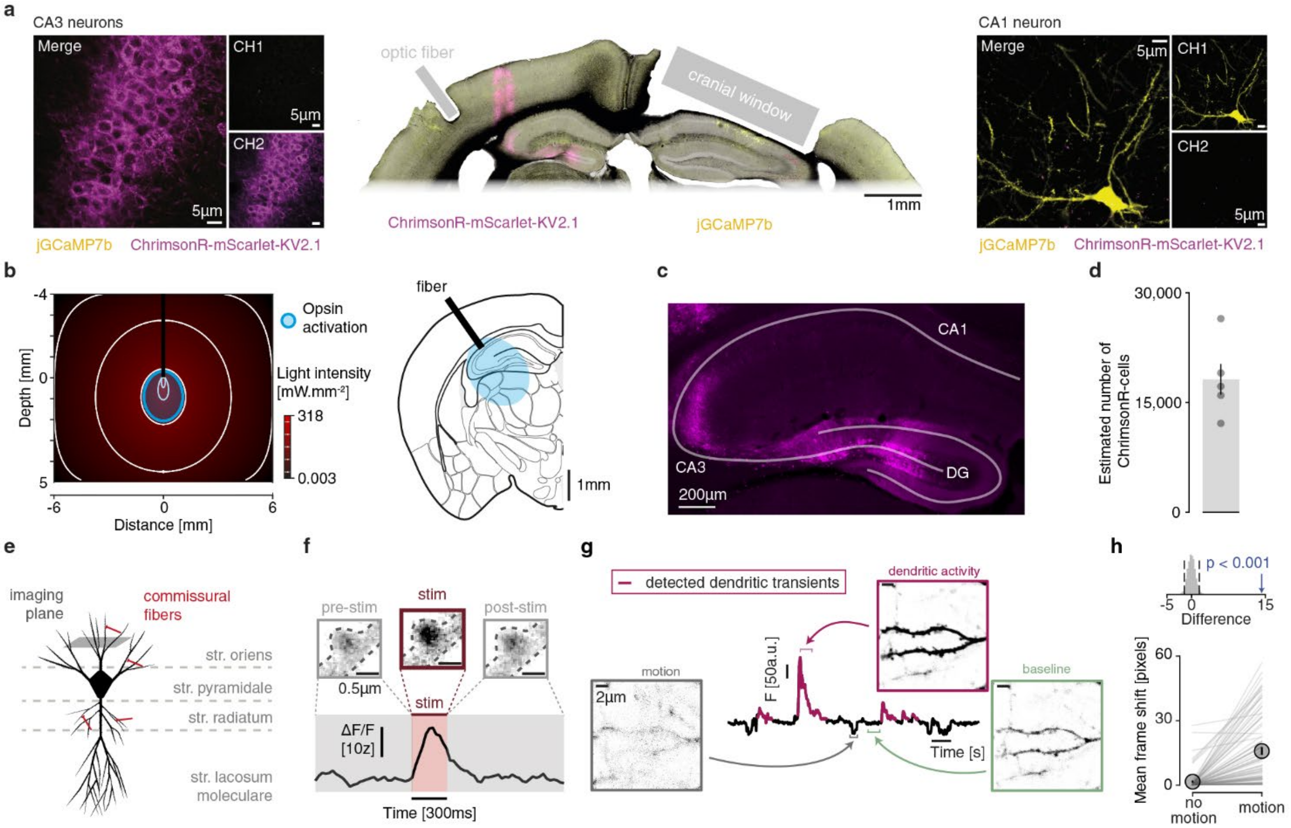

Figure S1. Imaging of spines in CA1.

a) Expression of soma-targeted ChrimsonR and jGCaMP7b. Left: clCA3 neurons expressing ChrimsonR-mScarlet-KV2.1 (magenta, CH1). Right: ilCA1 neurons sparsely expressing jGCaMP7b (yellow, CH2). Middle: Macroscopic overview of a brain section showing positions of optic fiber for stimulation above clCA3 and the chronic imaging window above ilCA1. **b)** Monte-Carlo simulation of light propagation from the tip of the optic fiber in the mouse brain. The area which was illuminated with sufficient irradiance to activate ChrimsonR (blue outline, left) is depicted in blue as an overlay on a section from the Allen Reference Atlas – Mouse Brain (right). Allen Mouse Brain Atlas, atlas.brain-map.org [1]. **c)** Confocal image of a brain section from contralateral hippocampus showing expression of ChrimsonR-mScarlet-KV2.1. **d)** Total number of cells in CA3 expressing ChrimsonR, estimated from post-hoc histology. Each datapoint represents one mouse (n = 5 mice). **e)** Schematic drawing of an ilCA1 neuron showing the imaging plane in stratum oriens. **f)** Example calcium trace of a single EPSCaT with corresponding t-projections of the spine before, during and after optogenetic stimulation. **g)** Example trace of dendritic fluorescence including bouts of locomotion. Calcium events are marked in magenta. Average time-projections are shown for baseline fluorescence (right, green) and for a dendritic calcium transient (magenta, top right). Motion events led to out-of-focus signal (left inset, gray frame). **h)** Quantification of frame shift during immobility and motion. Top: Permutation test. Arrow represents the true difference. Source data are provided as a Source Data file.

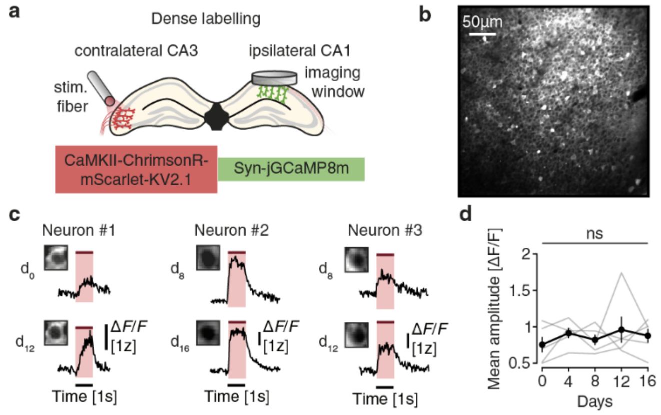

Figure S2. Stable optogenetic stimulation of ilCA1 over the time course of the experiment.
a) Schematic illustration depicting the injections and chronic implants for dense labelling of ilCA1 with jGCaMP8m. **b)** Example field of view with densely labelled neurons in ilCA1. **c)** Average postsynaptic calcium responses from three example neurons at two timepoints (d0 and d12). **d)** Average mean amplitude of postsynaptic calcium responses over the time course of the experiment for all responding neurons (n = 5 mice, 387 neurons). Linear Mixed Model with dendrites as random effect. ns: non-significant. Mean ± s.e.m. Source data are provided as a Source Data file.

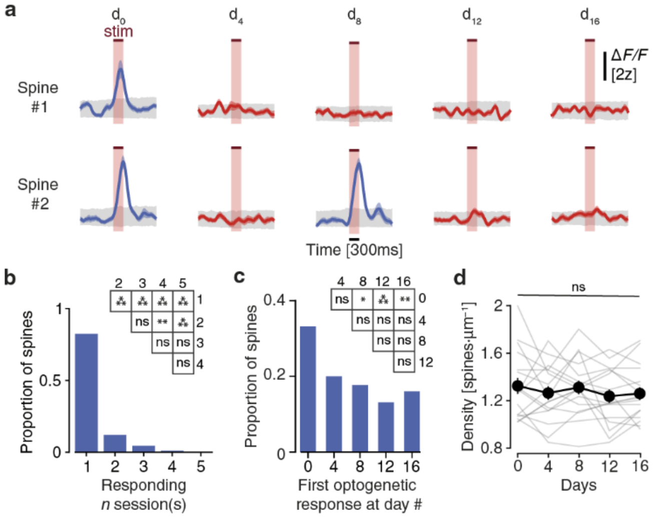

Figure S3. The majority of responsive spines show EPSCaTs only in one session.
a) Examples of response status changes over time. Spine #1 was responding in one session (d0) while spine #2 was responding in two sessions at days 0 and 8. Mean ± s.e.m. **b)** Distribution of spines according to the total number of sessions in which they showed a response. Pairwise chi- square tests are shown in the table. **c)** Distribution of spines according to the session in which they showed a response for the first time. Pairwise chi-square tests are shown in the table. **d)** Average spine density on all recorded dendrites. Linear Mixed Model with dendrites as random effects. *: $p < 0.05$, **: $p < 0.01$, ***: p < 001, ns: non-significant. Mean ± s.e.m. Source data are provided as a Source Data file.

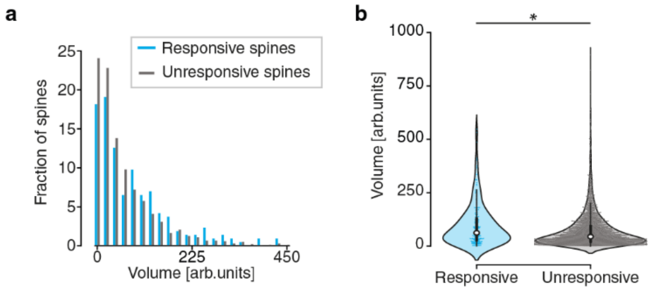

Figure S4. The volume of responsive spines is larger than the volume of unresponsive spines.
a) Histogram of the distribution of the volume of responsive (blue) and unresponsive (gray) spines. Same data as in Figure 2c. 22 equally spaced bins (20 arb. units) were used. **b)** Volumes of all responsive and unresponsive spines. Permutation tests. *: p < 0.05. Source data are provided as a Source Data file.

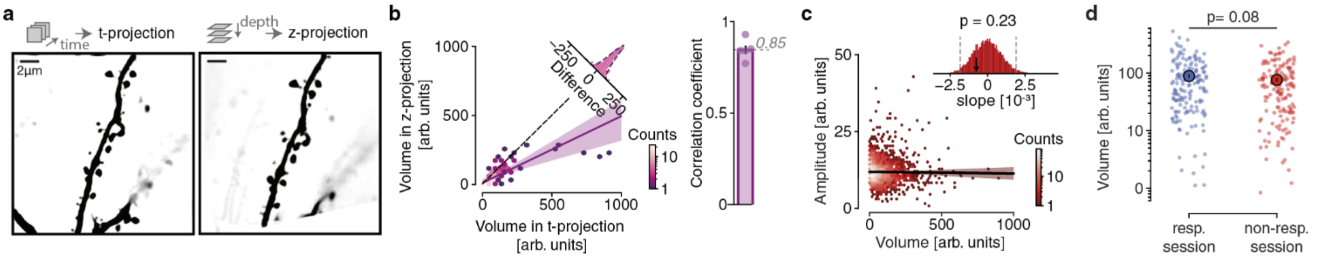

Figure S5. Spine volume estimation based on time series is independent of calcium activity.
a) Example projections of the same dendrite from a time-series (t-projection) and from a vertical stack (z-projection). **b)** Volume estimated from z-projections as a function of the volume estimated from t-projections for all spines (n=164 spines, 4 dendrites, 5 sessions). The high correlation coefficient indicates that both methods yield similar volume estimates. **c)** Spine head volume as a function of EPSCaT amplitudes in the respective spines. Absence of correlation indicates that EPSCaTs and spine volume can be measured independently. Permutation tests. **d)** Volume of responsive spines separately plotted for responding and non-responding sessions. Linear Mixed Model with spines as random effect. N= 175 responsive spines, 17 dendrites, 5 mice. ***: p < 0.001, ns: non-significant. Source data are provided as a Source Data file.

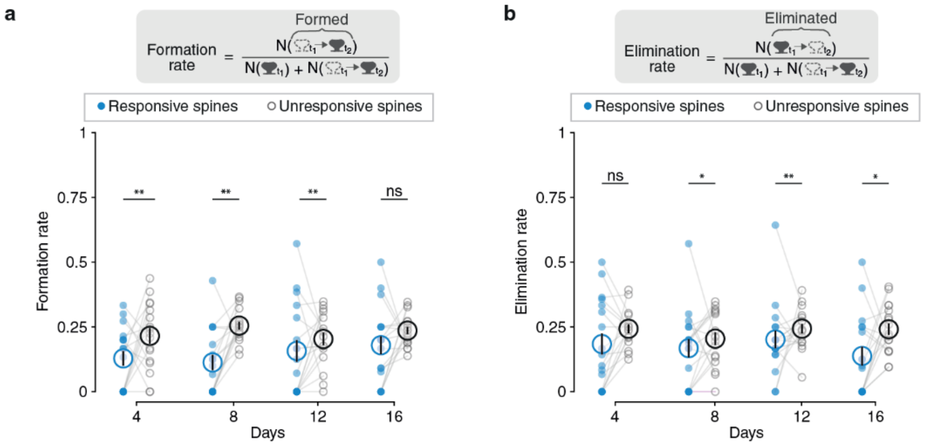

Figure S6. Both formation and elimination rates are different between responsive and unresponsive spines.
a) Formation rate and **b)** elimination rate of responsive spines versus unresponsive spines over time. Linear Mixed Model with dendrites as random effect and pairwise comparisons with Bonferroni correction. Mean ± s.e.m. N=175 responsive and 754 unresponsive spines, 17 dendrites, 5 mice. Source data are provided as a Source Data file.

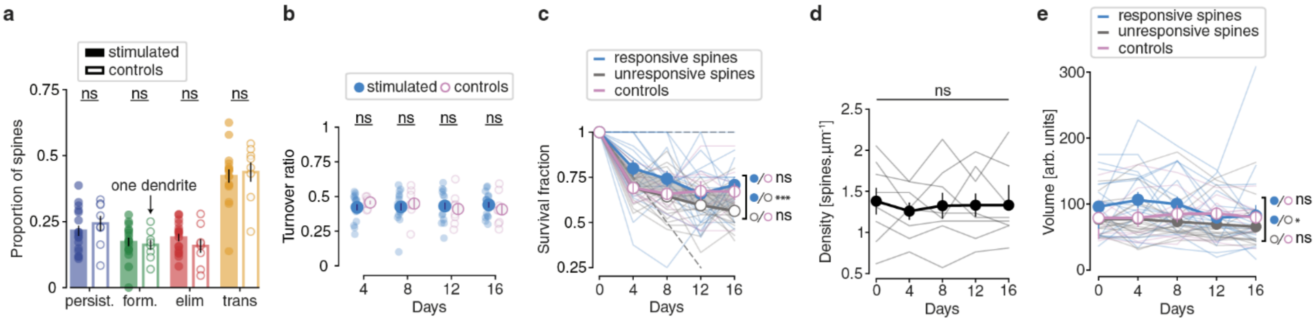

Figure S7. Optogenetic stimulation does not affect the overall stability or volume of dendritic spines.
a) Proportion of different dynamic spine categories on dendrites in control animals (no optogenetic stimulation, n=8 dendrites) and optogenetically-stimulated animals (n=17 dendrites). Linear Mixed Model and pairwise comparisons with dendrites as random effect. Mean ± s.e.m. Each dot represents one dendrite. **b)** Turnover ratio of stimulated dendrites and non-stimulated controls over time. Linear Mixed Model and pairwise comparisons with dendrites as random effect. Mean ± s.e.m. **c)** Survival fractions of control spines from non-stimulated animals and responsive and unresponsive spines from stimulated animals. Linear Mixed Model with dendrites as random effects and responsive, unresponsive and control as fixed effects. Mean ± s.e.m. **d)** Density of spines from control animals. Linear Mixed Model with dendrites as random effects. Mean ± s.e.m. **e)** Volumes of control spines from non-stimulated animals and responsive and unresponsive spines from stimulated animals. Linear Mixed Model with dendrites as random effect and pairwise comparisons. Mean ± s.e.m. ***: $p < 0.001$, *: $p < 0.05$, ns: non-significant. Source data are provided as a Source Data file.

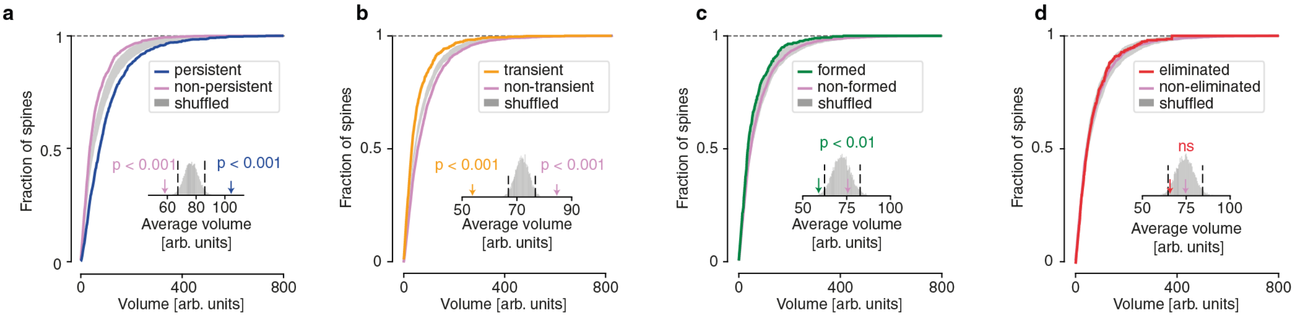

Figure S8. Stable spines have larger head volumes.
a) - d) Cumulative distributions of the head volumes of persistent (a), transient (b), formed (c) and eliminated (d) spines versus spines of all other categories (pink). Shuffled volumes are indicated by shaded gray area. Permutation tests. Arrows indicate actual average spine volumes of the respective categories. ***: $p < 0.001$, **: $p < 0.01$, ns: non-significant. Source data are provided as a Source Data file.

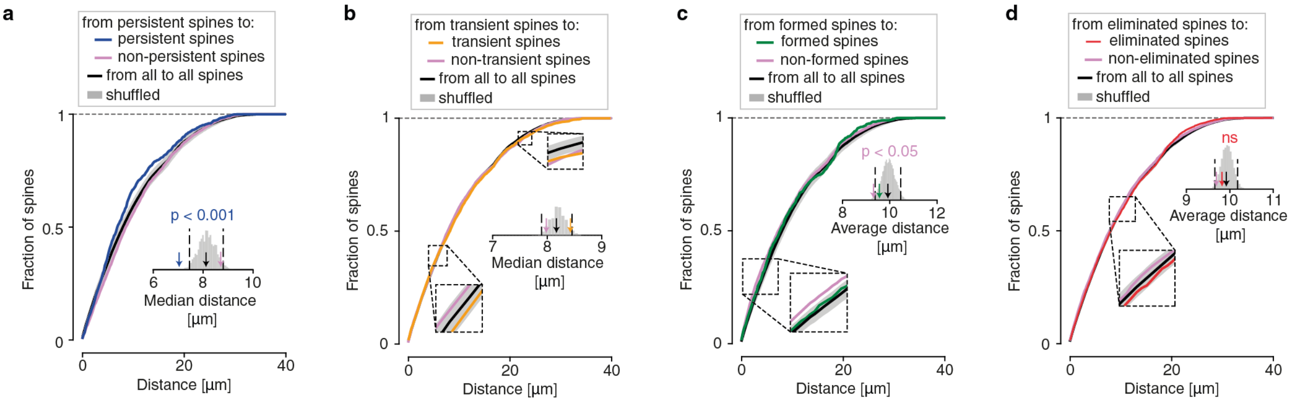

Figure S9. Persistent spines show higher proximity to each other than non-persistent spines.
a) - d) Cumulative distributions of distances between persistent (a), transient (b), formed (c), and eliminated (d) spines, compared to the distances to the other spines. Shuffled distances are indicated by shaded gray area. Arrows indicate actual average distance between spines of the respective categories. Permutation tests. ***: $p < 0.001$, *: $p < 0.05$, ns: non-significant. Source data are provided as a Source Data file.

Supplemental References

1. Allen Reference Atlas – Mouse Brain, Adult, 3D Coronal. Available from atlas.brain-map.org.
